# Vaginal Challenge with an SIV-Based Dual Reporter System Reveals That Infection Can Occur throughout the Upper and Lower Female Reproductive Tract

**DOI:** 10.1371/journal.ppat.1004440

**Published:** 2014-10-09

**Authors:** Daniel J. Stieh, Danijela Maric, Z. L. Kelley, Meegan R. Anderson, Holly Z. Hattaway, Beth A. Beilfuss, Katharina B. Rothwangl, Ronald S. Veazey, Thomas J. Hope

**Affiliations:** 1 Northwestern University, Feinberg School of Medicine, Department of Cellular and Molecular Biology, Chicago, Illinois, United States of America; 2 Tulane National Primate Research Center, Division of Comparative Pathology, Covington, Louisiana, United States of America; Emory University, United States of America

## Abstract

The majority of new HIV infections occur in women as a result of heterosexual intercourse, overcoming multiple innate barriers to infection within the mucosa. However, the avenues through which infection is established, and the nature of bottlenecks to transmission, have been the source of considerable investigation and contention. Using a high dose of a single round non-replicating SIV-based vector containing a novel dual reporter system, we determined the sites of infection by the inoculum using the rhesus macaque vaginal transmission model. Here we show that the entire female reproductive tract (FRT), including the vagina, ecto- and endocervix, along with ovaries and local draining lymph nodes can contain transduced cells only 48 hours after inoculation. The distribution of infection shows that virions quickly disseminate after exposure and can access target cells throughout the FRT, with an apparent preference for infection in squamous vaginal and ectocervical mucosa. JRFL enveloped virions infect diverse CD4 expressing cell types, with T cells resident throughout the FRT representing the primary target. These findings establish a new perspective that the entire FRT is susceptible and virus can reach as far as the ovary and local draining lymph nodes. Based on these findings, it is essential that protective mechanisms for prevention of HIV acquisition must be present at protective levels throughout the entire FRT to provide complete protection.

## Author Summary

There is currently a great effort world-wide to develop interventions such as vaccines and microbicides to decrease, or hopefully block, HIV transmission. To model the infection of women, the field utilizes the rhesus macaque vaginal transmission model. Understanding the initial events leading to infection after viral challenge of the female reproductive tract (FRT) is crucial for the development of functional prevention strategies. To this end, we developed a novel method for detecting infection in the rhesus macaque FRT after vaginal inoculation. This method utilizes single round replication defective vector that expresses dual reporter proteins, Luciferase and mCherry. Monitoring Luciferase expression allows us to identify the sites of infection within the intact FRT, while fluorescent protein mCherry allows us to visualize the single infected cells. Our studies revealed that virus can access the entire upper and lower reproductive tract. Infection occurs primarily in vaginal and ectocervical tissue, but can spread as far as the ovary and local draining lymph nodes. All classically defined susceptible cell types can be infected with the broadly tropic HIV envelope utilized in this study. Prevention strategies aimed at protecting from HIV infection should consider the entire FRT architecture as potentially susceptible and design interventions accordingly.

## Introduction

The majority of new female human immunodeficiency virus (HIV) infections are the direct result of vaginal intercourse with an infected male partner [1]–[3]. Previous studies looking at early transmission events have focused on the endocervix within the female reproductive tract (FRT) in part due to the preconception that these are preferential sites for transmission and in part due to the technical and time constraints [4]. Here, we describe methodology that allows us to survey the entire FRT for potential sites of infection and then characterize the initial target cells in a systematic and efficient manner using a rhesus macaque (*Macaca mulatta*; RM) SIV vaginal transmission model.

Current paradigms imply that a small number of initial transmission events occur, which subsequently rely on the host immune response to infection to fuel viral spread [4], [5]. The evidence that in most infections a single viral variant establishes systemic infection reinforces this model [6]. This is thought to occur in areas in the FRT that are particularly susceptible to viral entry. Multiple studies examining viral acquisition after vaginal exposure in macaque models have concluded that the endocervix and it's less robust single cell columnar epithelial barrier is a primary portal of entry for virions in the FRT [7], [8]. They have also suggested that the transformation zone, where the columnar epithelium of the endocervix merges with the more robust squamous epithelial barriers of the vaginal vault is especially susceptible because of inefficient barrier function where the two different types of epithelium come together. Unfortunately, these studies presented the results in a qualitative manner making it impossible for the reader to know how commonly infection in the endocervix/transformation zone was detected. In these papers it was also suggested that areas in the vaginal vault where squamous epithelial is dysfunctional might also be a site of acquisition in the FRT. Breaks in the vaginal epithelium due to abrasion or ulcerative STIs, or exposure of the more permissive simple columnar epithelium in the ectocervix due to cervical ectopy, can facilitate viral ingress [3]. These cervix-centric studies have led to the field focusing on this as a principle site of transmission and excluding much of the FRT as important to protect [7], [8]. Another implication of this work is that the primary bottleneck for transmission is at the mucosal barrier. Based on these observations it was concluded that preventive modalities, either microbicidal or vaccine based, present in the vaginal vault and endocervix would be sufficient to prevent transmission.

**Figure 1:**
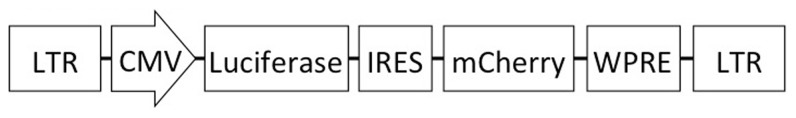
Schematic of the lentiviral vector used in this study. The vector contains an effLuc expression cassette, expression of which is driven by the CMV promoter. An internal ribosomal entry site (IRES) and a mCherry cassette are downstream of luciferase. This vector contains the SIV promoter in the 5′ long-terminal repeat (LTR) for efficient virus production in the context of a Tat-free packaging system, and self-inactivating mutations in the 3′ LTR.

To date, the only clinically demonstrated efficient prevention mechanism is based on oral pre-exposure prophylaxis (PrEP) that results in systemic distribution of antiviral drugs [9]. In contrast, topically applied antiretroviral gels in the FRT have only shown partial or no protection [10], [11]. Poor adherence in efficacy trials has complicated the determination of their efficacy [12], [13]. The disparity may reveal more about the mechanism of viral acquisition than was previously appreciated. Here we consider the possibility that there are more widespread infection events, including the upper FRT and local draining lymph nodes, which have been absent in previous studies on transmission. To this end, we have modified an SIV-based gene delivery vector [14] into a dual reporter system that expresses the mCherry fluorescent protein [15] and enhanced firefly luciferase [16] (Figure 1). This system expresses no viral proteins and thus only undergoes a single round of infection. By examining the entire FRT of animals 2 days after vaginal challenge, we find these initial acquisition events to be widespread.

## Results

### Vector design and optimization

The reporter system we use in these studies is designed to examine the localization and phenotype of cells that are susceptible to infection by the initial viral inoculum. We use a non-replicating vector that allows determination of the initial targets of infection, because any transduction events detected must be a derived from the challenge inoculum. Gene delivery through such a vector is commonly described as transduction, rather than infection because it only encompasses the replication steps up to the integration of the reporter genome. The reporter genome is delivered by a SIV-based lentiviral delivery system (Figure 1) which is necessary to avoid species-specific restriction factors like TRIM5α that are known to block HIV infection in rhesus macaques (RM). Use of two reporters provides complementary evidence for successful transduction. The luciferase reporter enables examination of whole tissues to find foci of infection with an *in vivo* imaging system (IVIS). Luminescence is used as macroscopic guide for detection of tissue sites where individual infected cells can be subsequently identified with fluorescence microscopy of cryosections.

The vector strategy allows different envelopes to be utilized to determine vector tropism. Here we compare HIV-1 envelope (strain JRFL [17]) or VSV-G protein mediated delivery of the vector. JRFL was chosen to be the initial HIV envelope used to develop this system for multiple reasons. It is CCR5 tropic, as is the virus that is sexually transmitted and has a broad tropism, able to infect a wide range of immune cells including T cells, macrophages, and dendritic cells. But perhaps most importantly, it is very efficient at pseudotyping this SIV vector allowing maximal titers of virus to be generated. Using VSV-G envelope enables exploration of barrier function and viral particle distribution throughout the FRT, because VSV-G is capable of transducing nearly all cell types [18]; JRFL envelope identifies sites of HIV susceptible cells.

The reporter particle genome does not express any viral proteins and instead encodes enhanced firefly luciferase [16] and mCherry fluorescent protein [15] from a single transcript. An internal ribosome entry site (IRES) is located upstream of the mCherry open reading frame to insure efficient expression of the downstream protein in this bicistronic message. Expression is driven by the immediate-early CMV promoter to optimize reporter gene transcription. A Woodchuck Hepatitis Virus post-transcriptional regulatory element (WPRE) located at the 3′ end of the message enhances gene expression of the vector [19], [20]. The WPRE is commonly utilized in lentiviral vectors to obtain efficient expression of delivered genes [21].

Reporter virions are generated by transfection of 4 separate plasmids into 293T cells, producing virus particles that undergo all of the early stages of the SIV replication cycle up through integration of the viral genome. The reporter genome is transfected along with the viral protein expression plasmids, expressing all of the proteins necessary to package the reporter genome into a functional, single round replicating virus particle. SIV proteins are expressed using a modified SIV expression plasmid that does not express Env and has deletions of the 5′ and 3′ LTRs [14]. The vector delivering the reporter genes contains the elements necessary for efficient packaging and integration after reverse transcription. Importantly, the packaging construct does not contain any RNA packaging sites ensuring that only the reporter vector genome is packaged into assembling virions. Pseudotyping with a third plasmid for Env expression, either VSV-G or JRFL, determines the cellular tropism and entry mechanism that is utilized by reporter virions. Additionally, an HIV Rev expression plasmid is added to enable nuclear export of reporter genomes, and induce efficient expression of the SIV packaging construct, reporter virus genome, and HIV envelope.

This system enables a single round of replication of a SIV-based virions and allows the identification of cells that have integrated viral genomes, which we utilize here as the threshold for defining an infected cell. Dual reporter expression indicates that the cell was susceptible to entry by the pseudotyped envelope, and permissive to uncoating of the viral capsid, reverse transcription, nuclear import of the viral genome and integration of reporter DNA and expression of fluorescent and luminescent proteins.

To develop our transmission model, we addressed the sensitivity of the ability to detect luciferase signal with an IVIS whole animal imaging system. JRFL pseudotyped dual reporter vector was used to transduce RM activated PBMCs, which were then quantified to show transduction of 0.5 to 2% of cells, varying by experiment. Increasing numbers of transduced cells were then subcutaneously injected into nude mice followed by intraperitoneal administration of luciferin. Luminescent signal was then analyzed an *in vivo* imaging system (IVIS). This pilot study revealed that we could detect as few as 10 cells in close proximity to an injection site (Figure S1). This pilot study also revealed that we could not detect single transduction events, revealing an important limitation of this system. Therefore, this system can only reveal the localization of small foci of infection where multiple cells are expressing luciferase in the context of tissue.

### Macaque female reproductive tract has uneven susceptibility to viral entry

To initiate our development of a transmission model in rhesus macaques, we began with a VSV-G pseudotyped version of the vector, anticipating that the broad tropism would more readily generate a detectable luciferase signal. To increase the possibility of detecting a luciferase signal we utilized a vaginal biopsy, which we anticipated would provide a site of focused transduction and allow detection of signal because of the potential limitation of detection described above. The biopsy site would simulate the effects of microtrauma and generate a place where the vector could bypass mucosal barriers. Six RMs were atraumatically inoculated, vaginally and rectally, with high titer VSV-G pseudotyped reporter virions (TCID_50_ range: 10^5.4^–10^6.3^). Four of the animals were pretreated with Depo-Provera (DP) to increase the likelihood of finding transmission events [22], while two were normally cycling. 48 hours after inoculation the animals were sacrificed. The entire reproductive tract as well as inguinal and iliac lymph nodes were removed and soaked in d-Luciferin.

**Figure 2:**
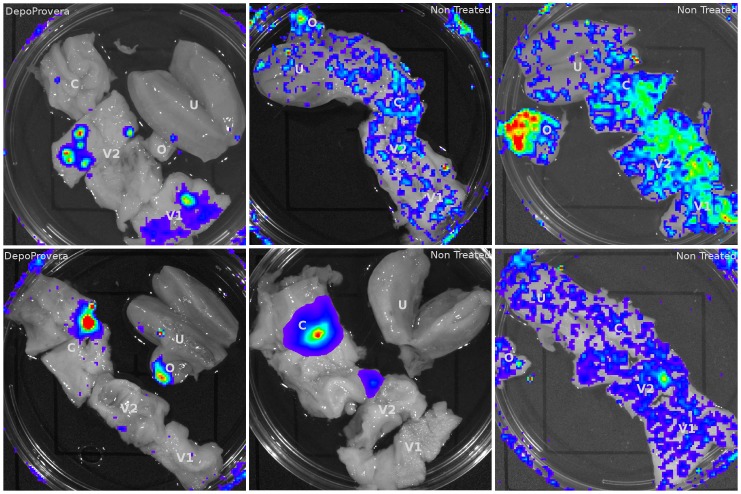
Luciferase reporter expression throughout the female reproductive tract in VSV-G pseudotyped vector inoculated Rhesus Macaques. 6 animals (4 pretreated with Depo-Provera) were inoculated with VSV-G pseudotyped dual-reporter vectors and sacrificed 48 hours later. Exposure to luciferin reveals reporter expression is present throughout the reproductive tract of Depo-Provera treated and normally cycling monkeys and that expression pattern varies between animals.

The initial analysis for luciferase activity delivered by the VSV-G pseudotyped vector revealed that virions could access viable cells throughout the entire FRT (Figure 2). The background signal from a naïve animal was used to establish the levels of specific luminescent signal (Figure S2). Luciferase activity was detected in the vaginal tissue, ecto- and endocervix, and ovaries. Between animals there was no uniform pattern of infection; although most had multiple foci of transduction present (Table 1). The effect of hormone treatment was not dramatic for the VSV-G pseudotyped vector and there was no evidence that DP treatment made a difference in the degree and localization of luminescence in this small study. The detection of luciferase activity in the ovary was unexpected. Luciferase activity was detected in small focal regions. These regions were dissected from the tissue and subjected to repeated rounds of assay for luciferase activity. Dissected regions that repeatedly demonstrated luciferase activity were frozen for cryosectioning and fluorescence imaging analysis to identify vector-transduced cells based on mCherry expression. In these initial studies we learned that dissecting the smallest possible piece of tissue (2×2 mm^2^) showing luciferase activity facilitated identification of single cells in thin tissue sections.

**Table 1:** Localization of luciferase expression in VSV-G pseudotyped LICh reporter inoculated Rhesus Macaques.

Distribution of infection throughout the FRT						
Animal	Hormone Treatment	Ovary	Vagina	Ecto-cervix	Endo-cervix	Lymph Node
**FM27**	Untreated	+	+			
**IB89**	Untreated	+	+			
**FM16**	Untreated	+	+			
**FM28**	Untreated		+	+	+	
**EI91**	DepoProvera		+			
**FM34**	DepoProvera	+	+	+	+	+

LICh reporter vector could reach viable cells throughout the reproductive tract and access local draining lymph nodes.

**Figure 3:**
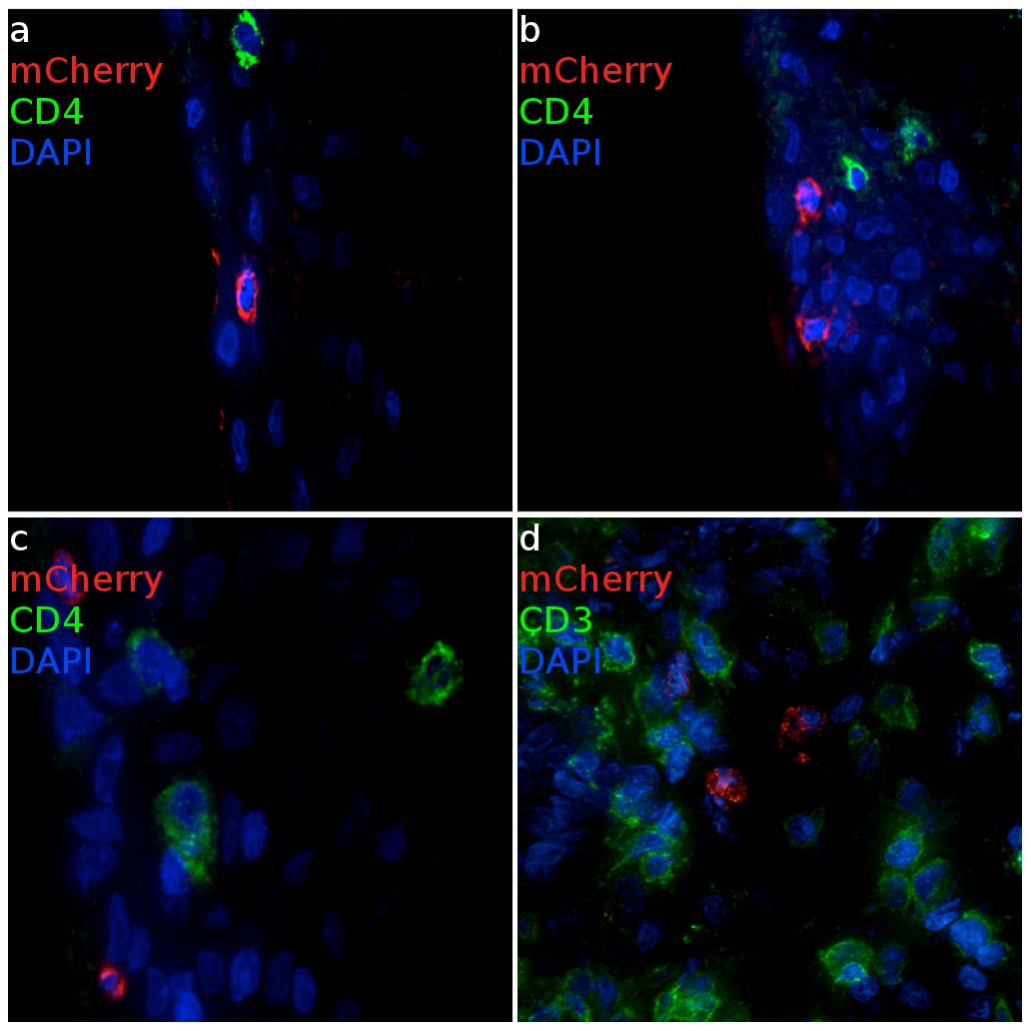
Infected cells from ectocervical and vaginal tissue of VSV-G enveloped reporter inoculated Rhesus Macaques. (a, b) Virus primarily infects epithelial cells within the ectocervix. CD4 expressing cells cluster proximal to mCherry expressing cells but are not infected. (Animal code: FM27) (c) Epithelial and (d) sub-epithelial cells in the vagina are also susceptible to infection. Where infection is found, there are abundant numbers of T cells are present. (Animal code: FM28) mCherry signal is shown in red. CD3 or CD4 is shown in green. Nuclei (DAPI) are shown in blue. Scale bars, 30 µm.

Expression of mCherry fluorescence revealed that epithelial cells in the ectocervix (Figure 3a, b) and vagina (Figure 3c) appear to be the primary target for VSV-G enveloped virions, although infection events below the basement membrane of squamous epithelium were observed in some cases and associated with a high density of T cells (Figure 3d). Cells that appeared to have strong emission through the mCherry filter set were assessed by two independent measures to confirm the transduction. The primary criteria to validate the presence of infection used spectral imaging, as mCherry has a narrow emission spectra peaking at 610 nm [15], a signature that is not known to exist naturally. Additionally, cryosections were stained for luciferase expression utilizing immunofluorescence with an anti-luciferase antibody, revealing that the mCherry expressing cells were also positive for luciferase expression from the dual reporter vector. To further characterize the signal, we determined fluorescence in the TRITC channel as a negative control. Therefore, cells that scored positive for both mCherry and luciferase expression were analyzed through a 586 nm TRITC filter set and were found to have dim emission, consistent with mCherry having low fluorescent signal at 586 nm. Weak relative emission in orange wavelengths indicates that the fluorescent signal seen is not a part of some broad-spectrum autofluorescent signal that can be detected in tissues and is more applicable to screening large numbers of cells than spectral imaging. Therefore, in many cases we utilized the TRITC signal as a surrogate of spectral imaging. The same criteria held true for confirming the infection of RM PBMCs (Figure S3).

Through pilot studies with VSV-G pseudotyped dual reporter vector we were able to demonstrate that we can detect vector expressed luciferase signals after vaginal exposure to virus. Importantly, we were able to optimize techniques to unequivocally identify mCherry expression of transduced cells by spectral imaging and luciferase staining in cryosections of small pieces of tissue exhibiting luciferase activity. Through this process we developed criteria to identify the bulk of transduced cells on a fluorescence microscope by 3 criteria: 1) mCherry positive, 2) luciferase positive after antibody staining, and 3) minimal TRITC fluorescence consistent with a signal from mCherry fluorescence rather than broad-spectrum tissue autofluorescence.

### HIV Env pseudotyped vector can transduce cells throughout the entire FRT

**Figure 4:**
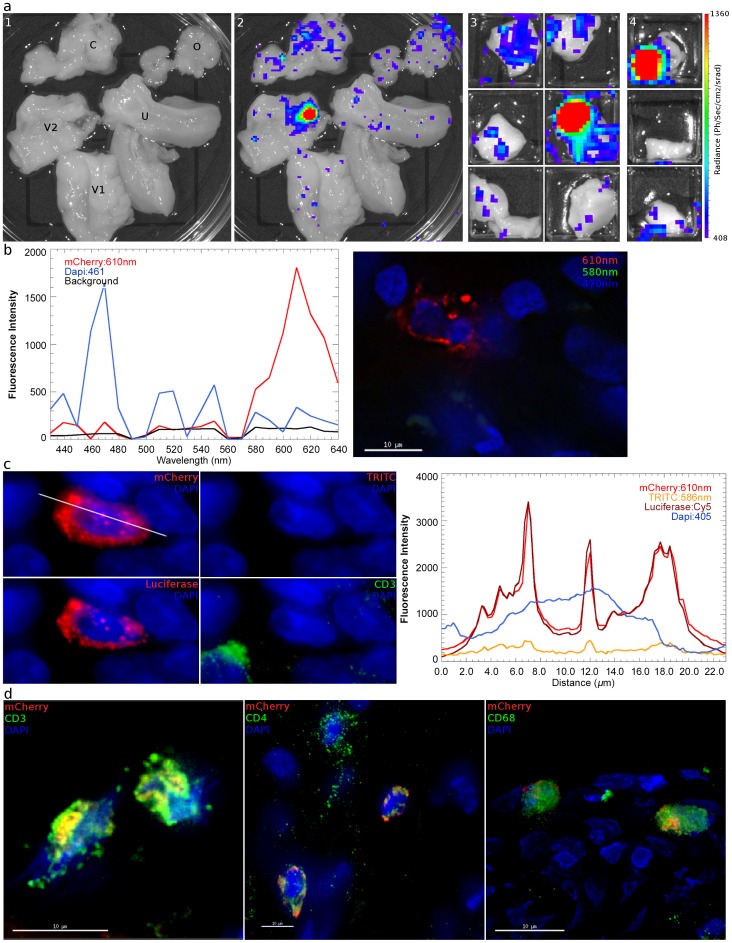
Identification and phenotyping of infected cells from female reproductive tract. a, 48 hours after inoculation with JRFL pseudotyped LICh vector, the reproductive tract is separated into large sections (panel 1; V1: labia and lower vagina; V2: upper vagina; C: cervix; U: uterus; O: ovary), soaked in d-luciferin to identify luciferase expressing regions with in vivo imaging systems, and dissected into sequentially smaller pieces (panels 2–4) to isolate strongly luminescent regions measuring 2×2 mm^2^. (animal code: EH99) **b,** Left panel; The spectral emission profile of a putative infected cell from the tissue identified in **a** is measured on laser scanning confocal microscope with resonant scanner. Emission profile matches the defined profile of mCherry. Right panel; Pseudo-colored reconstructed image on the right shows the localization of mCherry (red), DAPI (blue) and background signals (green). **c,** Vaginal tissue is stained for firefly luciferase expression. The combination of mCherry^+^ (red), TRITC^−/low^ (red), luciferase^+^ (red) fluorescence is criteria for confirming infection. The right panel shows relative fluorescence intensity of mCherry, TRITC, luciferase, and nucleus is along the line in mCherry image. (animal code: HP79) **d,** Phenotyping of infected cells by surface marker staining, shown in green, for CD3 (T Cell), CD4 (HIV-1 primary receptor), or CD68 (tissue resident macrophages) shows all conventionally defined HIV susceptible cell types are transduced within the vaginal tissue shown in **a**. (animal code: EH99).

Having validated the ability of the system to identify sites on infection with VSV-G pseudotyped vector, we next asked if the system would work after pseudotyping with an HIV envelope. We chose to utilize the JRFL CCR5-tropic envelope because of its broad tropism and ability to efficiently generate high titer vector. Concentrated stocks of JRFL Env expressing vector (TCID_50_ range: 10^4.8^–10^6.2^) were inoculated into eight RMs with vaginal biopsies; four animals were DP treated. During luciferase detection of these animals, dissection into subsequently smaller regions enabled focusing of the luminescent signal into foci (Figure 4a), which were frozen. The change in envelope did not result in noticeably decreased gross luminescent signal or an altered macroscopic pattern of infection. As observed with the VSV-G pseudotyped vector, luciferase activity in the ovary was also detected with the HIV envelope. Detectable levels of luciferase expression were identified within at least one ovary in six out of eight animals (two treated with DP, four untreated, Table 2).

**Table 2:** Localization of luciferase expression and microscopic confirmation of infection in 8 JRFL pseudotyped inoculated Rhesus Macaques.

Distribution of infection throughout the FRT											
Animal	Hormone Treatment	Luminescence Analysis					Microscopic Analysis				
		Ovary	Vagina	Ecto-cervix	Endo-cervix	Lymph Node	Ovary	Vagina	Ecto-cervix	Endo-cervix	Lymph Node
**GK26**	Untreated	+	+				+	+			
**DH39**	Untreated	+	+	+			+	+	+		
**IA80**	Untreated	+					+				
**HP79**	Untreated	+	+					+			
**CT82**	DepoProvera	+		+	+				+	+	
**FM09**	DepoProvera	+	+	+		+	+	+			+
**FF08**	DepoProvera		+	+		+		+			
**EH99**	DepoProvera		+	+				+	+		

All tissues collected are capable of harboring cells transduced by the initial inoculum.

Regions of the FRT expressing luminescent signal after vaginal exposure to JRFL pseudotyped vector were cryosectioned to identify the presence of infected cells by spectral imaging and fluorescence microscopy. The measured emission spectra of infected cells peaked at 609 nm (Figure 4b), confirming expression of the mCherry fluorescent protein in transduced cells. Immunofluorescence staining of tissue sections revealed that the mCherry expressing cells were also positive for luciferase expression. Because spectral imaging could not be applied to all potentially transduced cells, a surrogate of fluorescence spectra was achieved by measuring whether cells express a signal in the TRITC filters, which correspond to 586 nm (Figure 4c, d). In this manner, transduced cells were identified by 3 distinct imaging criteria. Infected cells were found within all JRFL inoculated animals. Tissues where luminescent signal could not be confirmed were typically due to high levels of background autofluorescence that prevented identification of mCherry expression.

We then examined the cellular phenotype of the JRFL pseudotyped reporter transduced cells by staining for CD4 (HIV-1 receptor), CD3 (T cell specific) or CD68 (macrophage specific). We find that both CD4 T cells and macrophages are capable of being infected within the same vaginal domain (Figure 4d). The JRFL pseudotyped vector universally transduced cells expressing CD4, although expression levels vary. This is likely a consequence of the known variability of CD4 expression between monocytic lineages and lymphocytes [23]. Additionally, activation status can also influence CD4 expression levels. CD3 expression was present on the majority of target cells, indicating that T cells were the preferred targets of transduction, even though the JRFL Envelope is defined as macrophage-tropic (Table 3). A similar preference for infection of T cells was observed whether the animals received Depo-Provera treatment or not. Considering transduced cells across all 8 JRFL inoculated animals, T cells were the dominant targets, representing 71% of all transduced cells. A smaller subset of infected cells was found to express CD68 revealing that tissue resident macrophages can also be transduced after vaginal inoculation. Non-specific primary antibody and secondary antibody only controls were used to establish the specificity of each cell marker used for phenotyping (Figure S4).

**Table 3:** Phenotype of SIV infected cells after inoculation JRFL pseudotyped virions.

Hormone Treatment	Infected Cell Phenotype			
	CD4+	CD4−	CD3+	CD3−
Untreated	**78 (100)**	**0 (0)**	**26 (68.4)**	**12 (31.6)**
DepoProvera	**49 (100)**	**0 (0)**	**64 (72.7)**	**24 (27.3)**
Total	**127 (100)**	**0 (0)**	**90 (71.4)**	**36 (28.6)**

Antibody labeling of CD3 and CD4 cells was used to determine infected cell phenotype. Total cell number and relative % are reported.

**Figure 5:**
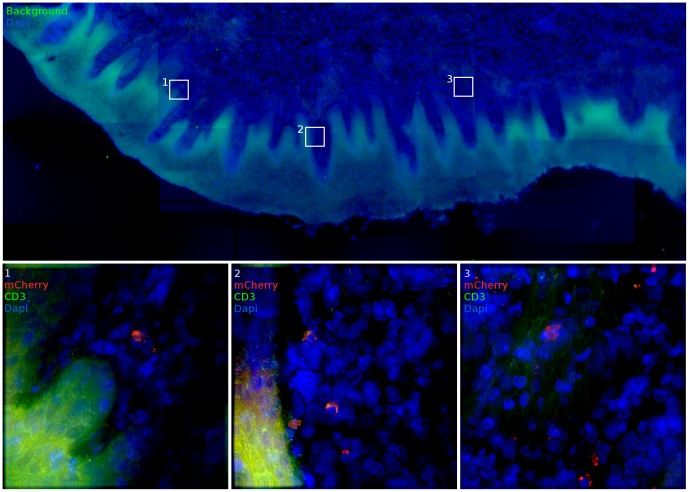
Vaginal tissue with intact epithelium is the most common target for target cell transduction. 48 hours post JRFL pseudotyped vector administration, the FRT was removed and luminescent foci identified by IVIS analysis (inset). In vaginal domains without biopsy, the stratified epithelium is intact. In these portions of the vaginal vault, infected cells can still be found. mCherry expression (red), CD3 staining (green) and nuclei label (blue) are used to show transduced cells. (animal code: GK26).

Throughout the vaginal vault, where viral challenge was administered, transduced cells were consistently located in the lamina propria below the basal epithelium (Figure 5). The ectocervix and vagina were the most common location where infected cells were found, with foci identified in all but one animal challenged with JRFL pseudotyped vector. Luminescent signal and microscopic identification of transduced cells were generally the strongest at biopsy sites, where virions could bypass mucosal barriers. However, biopsy sites represented only a minority of the foci identified within the vaginal vault. In regions where the stratified epithelium was intact, distal from a biopsy, transduction events were found in both T cells (Figure 5, Region 2) and non-T cells (Figure 5, Regions 1, 3). Reporter virions are therefore able to penetrate the squamous epithelium and transduce susceptible cells resident below an unbroken vaginal epithelium. The epithelial thickness and lack of a keratinized epithelium in this animal is consistent with previous descriptions of the RM vaginal epithelium in the luteal phase of the menstrual cycle [24].

**Figure 6:**
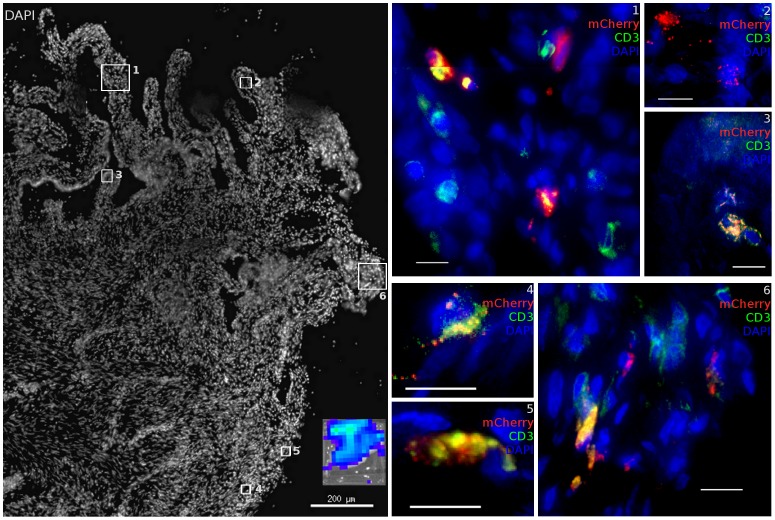
Diverse cell types are infected within the cervix. Within the cervix, both endocervical (Region 1–3) and ectocervical (Region 4–6) domains in the area of the transformation zone contain many resident T cells which are susceptible to infection with JRFL pseudotyped LICh vector. Non-T cells are also infected. Inset shows luciferase expression within dissected tissue before freezing. Scale bars measure 10 µm unless otherwise specified. mCherry expression (red), CD3 staining (green) and nuclei label (blue) are used to show transduced cells. (animal code: CT82).

Further into the FRT, we find infection was also possible within the endocervix, consistent with previous studies that have focused on this site. Only 1 out of the 8 animals had evidence of infection by luminescence analysis of endocervical tissue. However, in animal CT82, a focus containing many transduced cells was identified (Figure 6). The luminescent signal from this tissue was modest, potentially due to mucus produced in the endocervical crypts preventing efficient diffusion of luciferin. It is also possible that foci of infection are rare in the endocervix. Both T cells (Regions 1, 3, 4, 5, 6) and other target cells (Regions 1, 2, 6) could be infected in close proximity. The endocervix had many T cells present, with only a simple columnar epithelium separating them from the luminal surface. The transformation zone, where the epithelial architecture changes from stratified epithelium of ectocervical tissue (Regions 4–6) to simple columnar epithelium of endocervical tissue and the upper FRT (Regions 1–3) did not have a uniquely dense population of target cells transduced relative to other regions identified throughout the FRT where transduction occurred. No breaks in the epithelium were present in the focus of infection within the endocervix.

**Figure 7:**
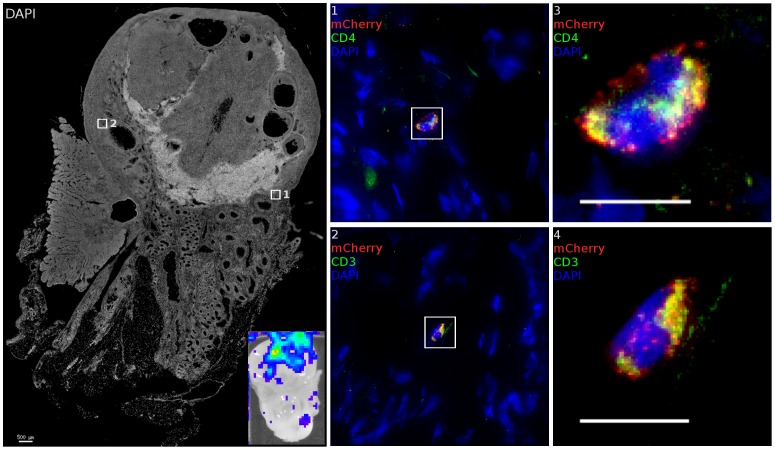
The ovary is susceptible to infection. After inoculation with JRFL pseudotyped LICh vector, tissues demonstrating luminescent signal (inset) is stained for luciferase and CD3 or CD4. Inset shows luciferase expression within tissue before freezing. Transduced cells within each region are shown as numbered close-up. Infected cells are found within the cortical zone of the ovary. Infected cells are not found within developing ova. The cell in region 1 and 3 expresses HIV-1 receptor CD4. Staining the adjacent tissue section identifies the transduced cell in region 2 and 4 as being CD3^+^. (animal code: GK26).

Although no luminescent signal was detected in the uterus or oviduct, we did find that virus was able to localize to and transduce cells in the ovary. A luminescent signal was identified in ovarian tissue of 6 out of 8 animals. When ovarian tissue was stained for CD3, 79% of transduced cells found within the ovary are found to be T cells, a further bias towards infection of T cells than is seen on the whole of the FRT. Transduced cells were primarily present in stromal tissue of the cortical zones of the ovary, proximal to developing follicles although not seen in *corpus albicans* structures. The high density of blood and lymphatic vessels in the helus does not have a corresponding increase in the density of transduction. As seen in Figure 7 regions 1 and 2, close proximity to a blood vessel was not associated with transduced cell localization; the helus and medullary zone rarely had any transduced cells present. More detailed imaging of these cells (Regions 3, 4) shows a typical lymphocyte morphology associated with the infected T cells. Furthermore, RMs did not have to be experiencing a regular menstrual cycle or have recently ovulated for virions to access the ovary, as animal FM09 was treated with Depo-Provera and still had transduced cells identified. 3 of the 4 regularly cycling RMs had transduction events as confirmed by identification of mCherry positive single cells. T cell clustering at the sites where foci of infection, as was found in the lower FRT, did not take place to the same extent in the ovary. Instead, more diffuse distribution of transduction occurred, with transduced cells found over regions spanning the diameter of the ovary. It is not clear if this is due to higher levels of T cell migration or distinct foci of transduction.

Within local draining lymph nodes, only infrequent evidence of infection is found. Two animals were identified with modest luminescent signal in inguinal lymph nodes. Further examination revealed a number of CD4 cells expressing mCherry and luciferase reporters (Figure S5), but high levels of auto-fluorescent background limited the scope of examination.

To validate our findings of mCherry and luciferase reporter expression we utilized nested PCR designed to detect rare transduction events within the tissue of exposed animals. Such PCR based approaches have been previously utilized to find evidence of rare infections events within days of vaginal or oral exposure of SIV [25], [26]. Therefore, we conducted nested PCR on tissue from luciferase positive and negative tissues within the FRT of challenged animals. Tissues from unexposed animals were utilized as a negative control. Tissue was sectioned in the same manner as for fluorescence imaging, and DNA was extracted from the equivalent of 200 µm of tissue. 250 ng aliquots of input genomic DNA, corresponding to approximately 3.8×10^4^ cells, was added per PCR reaction and at least 24 reactions were performed per tissue extract. All final products were separated on agarose gels and visualized by ethidium bromide staining. Amplification of the mCherry gene was confirmed by extracting DNA from the gel and sequencing with the inner set of primers.

**Figure 8:**
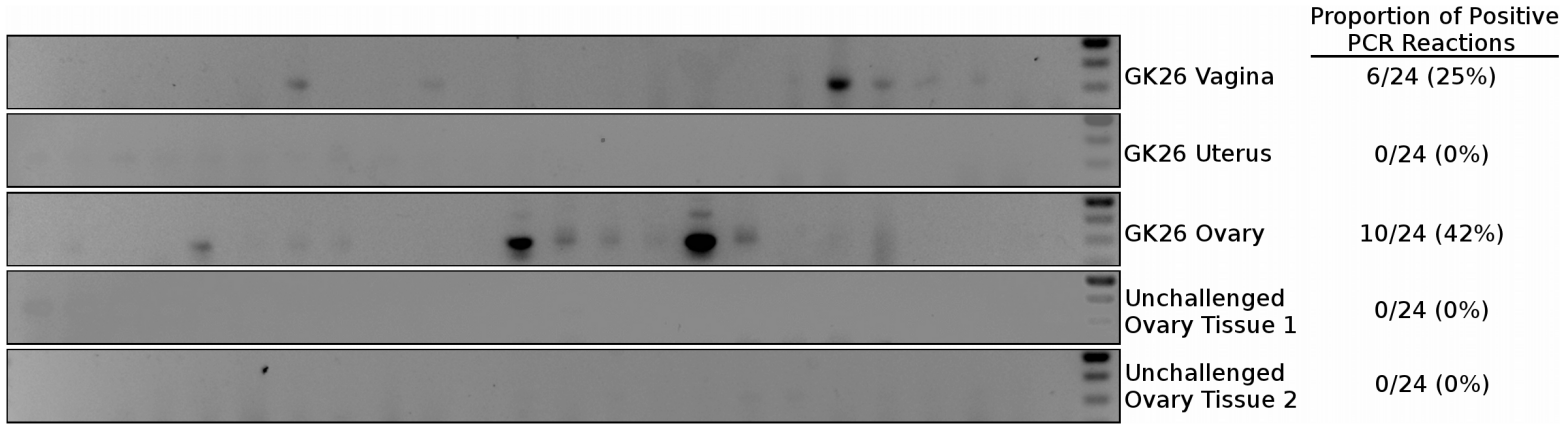
Nested PCR of macaque tissues. Distribution of LICh reporter DNA-positive tissues in the female reproductive tract after inoculation with JRFL pseudotyped LICh vector. Results are based on the number of mCherry positive reactions, with at least 24 reactions per experiment. Gel shown is representative of three independent DNA extractions and amplifications.

Tissues that had a detectable luciferase signal corresponded to those in which there was a positive PCR signal (Figure 8). The ovary of a LICh inoculated animal that did show considerable luminescent signal in that tissue (GK26) contained multiple copies of mCherry DNA. Importantly, genomic DNA extracted from the ovaries of 2 animals not treated with the LICh virus showed no evidence of DNA encoding the mCherry gene. Therefore, the luminescent and mCherry fluorescence signals correlated with the presence of reporter DNA revealing the ability of luciferase signal to reveal sites of transduction. In contrast, DNA from uterine tissue of the same animal which showed no luciferase signal also showed no evidence of transduced DNA as detected by nested PCR. The ability of the luciferase signal to identify DNA positive tissue further validates the two-reporter vector system to identify sites of vector transduction after vaginal exposure in the rhesus macaque model. It is important to note that the PCR method also validates the unanticipated detection of transduced cells within the ovary, while apparently bypassing the uterus.

To summarize, the regions of the FRT where luciferase and mCherry expressing cells were detected in the JRFL pseudotyped vector exposed RMs, varied between animals and could be identified throughout the FRT and could extend to local draining LNs (Table 2). The majority of animals had more than one site of transduced cells, revealing that there are typically multiple sites where the virus can gain access to potential target cells at the time of exposure in the rhesus macaque model. The primary site of infection was within the vaginal vault with seven of the eight animals having sites of infection identified in the vagina and/or ectocervix. The second most common site of the identification of transduced cells was the ovary. Transduced cells in the ovary were identified in half of the animals. Detection of transduced cells in the endocervix and local draining lymph nodes was rare, both being confirmed in a single animal.

## Discussion

The anatomical site and phenotype of the initial cells infected by immunodeficiency viruses within the FRT is heavily debated. Previous studies using similar high virus challenges have suggested that the endocervix is a primary site of infection in a RM vaginal challenge model [8], [26]. However, these studies were limited because they were not able to survey the entire FRT and surrounding lymphatic tissue for sites of infection. In contrast, using the dual reporter system developed here, which allows a survey of the entire FRT, we find a clear preference for infection of the squamous mucosal vaginal and ectocervical barriers of the vaginal vault with a high dose challenge, although only a small fraction of tissue contains transduced cells. When surveying the entire exposed tissue of the FRT two days after inoculation, the infection within the endocervix was rarely detected. The low frequency of transduction events detected in the endocervix might be due to the limitations of the system utilized here. Because of the protective mucus barrier coating the endocervical mucosa, foci of infection may be rare. However, transduction of disperse target cells may take place at a frequency that remains to be determined.

There are disparate models of the initial cell type of infection during male-to-female transmission, with some studies implicating CD4 T cells [26], [27], others implicating Langerhans cells [28], while others report mixed pools of target cells [29], [30]. Using the JRFL envelope, which has broad ability to infect different types of target cells in tissue culture experiments, we detect a preference for the infection of CD4 T cells in the different anatomical sites. Infection of CD4 T cells was detected approximately 70% of the time. However, the vector tropism observed here should be interpreted with caution because JRFL represents a lab adapted strain with a tropism of an HIV derivative isolated from the brain of a chronically infected individual, and may be biased away from the primarily T cell tropic nature of transmitted/founder viruses. Future studies will utilize envelopes from so called transmitted/founder viruses to gain insight into the initial targets of infection in the FRT. However, these initial observations reveal that a variety of immune cell types can be the targets of the initial inoculum of exposure during the transmission event.

While some have suggested that target cells in the upper FRT (uterus) might be sites of transmission [31], the possibility of infection of target cells in the ovary and lymph nodes after exposure has not been considered. It is known that potential target cells are present in the ovary and that hormonal changes associated with the menstrual cycle can modulate both the number and localization of immune cells. The ability of the virus to travel far enough to reach the ovary was unanticipated, but consistent with evidence that materials within the vaginal vault and uterus can migrate to the ovaries. For example, there is a known association between perineal talc use and ovarian cancer risk [32]–[34]. Additionally, retrograde menstruation through the fallopian tubes is commonly reported in females, even in the absence of endometriosis, but never associated with dissemination of pathogens [35], [36]. Likely the mechanism(s) behind this undefined retrograde transport is also influencing virus transport to the ovaries in this model. The ability of the ovary to be the portal of transmission remains to be defined. However, the studies presented here demonstrate that particles from a high titer vaginal inoculation can reach target cells present in this tissue.

The mechanism by which infected cells are present in the lymph node is not apparent; vaginal or rectal inoculation could lead to virions accessing draining lymph nodes. Because the kinetics of expression require a day post-entry for reporters to be detectable, *trans*-infection of resident T cells by migrating DCs carrying virions to lymph nodes is unlikely, though a possibility at later time points [37]. Migration of infected cells or virions directly entering the lymphatic drainage allows a sufficient time window for detectable reporter expression within lymph nodes after 48 hours.

The distribution of sites transduced by either the VSV-G or JRFL pseudotyped vectors was quite similar. Both were able to reach the ovaries, and typically were observed to breach mucosal barriers in discrete foci. Yet the two different vectors reflected very different and anticipated tropism. This indicates that the distribution of viral particles in the FRT after vaginal exposure is not strongly influenced by envelope tropism. Throughout most of the reproductive tract, the mucosal barriers functioned to exclude virions and prevent infection from occurring, but where the barriers were overcome, there were multiple cells being infected. It is also notable that T cells are abundant where infection occurs, irrespective of the envelope protein (Figure 3). Where mucosa is locally inflamed, such as a site of thinned or broken epithelium, increased numbers of immune cells are recruited [38], [39]. The large numbers of T cells seen where VSV-G pseudotyped virions have infected sub-basal cells are not recruited in response to viral replication, as only a single round of infection occurs. Instead, preexisting target cell clusters associated with functional defects in the mucosal barriers may represent sites of vulnerability.

The phase of the menstrual cycle was not controlled as a part of this study, but could play a significant role in viral acquisition [40], [41]. Animal GK26, which had infection in the vagina (Figure 5) and ovary (Figure 7), had a vaginal epithelial structure representative of the luteal phase. The lack of infection found in the uterus of this animal either by IVIS, or nested PCR is consistent with the lack of CD4+ T cells and macrophages in the uterine endometrium late in the menstrual cycle [38], [42], [43]. However, the possibility of rare transduction events occurring cannot be ruled out as it is not possible to test all uterine tissue for single transduced cells.

These findings on the wide range of locations and cell types susceptible to SIV have fundamental implications for development of prevention strategies. The sites of initial infection are not restricted to specific domains within the FRT tract and are not consistent between RMs. The observed abundant infection within the macaque vaginal vault is consistent with the failure of the diaphragm to protect women from HIV acquisition [44]. Further, we find a high density of target cells where virus enters the epithelium, suggesting that preexisting sites of local inflammation represent hotspots of vulnerability to infection by the initial inoculum. This is consistent with the known association of increased susceptibility to HIV infection with increased local inflammation. To better protect against female acquisition of HIV, it is important that vaccine or antiviral activity be widely distributed throughout the FRT architecture. The development of HIV preventions strategies should consider the upper FRT and resident vulnerable cell types as acquisition targets during male-to-female transmission.

## Materials and Methods

### Ethics statement

All animal studies were conducted in accordance with protocols approved by Northwestern University and Tulane National Primate Research Center local Institutional Animal Care and Use Committees (IACUC), protocol P0153. This study was carried out in strict accordance with the recommendations in the Guide for the Care and Use of Laboratory Animals of the National Institutes of Health (NIH) and with the recommendations of the Weatherall report; “The use of non-human primates in research”. All procedures were performed under anesthesia using ketamine hydrochloride, and all efforts were made to minimize stress, improve housing conditions, and to provide enrichment opportunities (e.g., objects to manipulate in cage, varied food supplements, foraging and task-oriented feeding methods, interaction with caregivers and research staff). Animals were euthanized by ketamine hydrochloride injection in accordance with the recommendations of the panel on Euthanasia of the American Veterinary Medical Association.

### Cell culture and infection

293T cells (American Type Culture Collection) were cultured in Dulbecco's modified Eagle's medium (HyClone) containing 10% fetal bovine serum, 100 Um L^−1^ penicillin, 100 µg mL^−1^ streptomycin and 292 µg mL^−1^ l-glutamine (Gibco). Rhesus macaque peripheral blood mononuclear cells (PBMC) were separated over Ficoll gradient and cultured at 2.5×10^6^ cells mL^−1^ in RPMI containing 10% fetal bovine serum, 100 U mL^−1^ penicillin, 100 µg mL^−1^ streptomycin and 292 µg mL^−1^ l-glutamine. 24 hours prior to infection with vector, cells were activated with 100 IU ml^−1^ IL-2 and 5 µg mL^−1^ Phytohaemagglutinin (PHA). 1000 TCID_50_ JRFL pseudotyped vector was added to activated PBMC. Infected cells were sorted for mCherry expression on a Beckman Coulter MoFlo system.

### Dual reporter vector and virus production

A SIV-based pseudovirus vector system was generated by modification of the SIV3 vector system [14]. The codon-optimized firefly luciferase gene is expressed through a poliovirus internal ribosome entry site (IRES) [16]. mCherry was chosen as it has unique narrow emission spectrum (610 nm) that is not known to exist naturally. The transcription of mCherry and luciferase reporters are driven from the constitutive immediate-early CMV promoter and their expression is stimulated by WPRE to facilitate robust expression. Vector design is shown in Figure 1. Pseudotyped reporter virus is produced by transfection of 293T cells with 4 plasmids complexed with Polyethyleneimine (PEI, Polysciences): reporter described above, SIV3+ packaging vector, REV expression plasmid DM121 and either a VSV-G envelope or JRFL (HIV-1 CCR5 tropic) envelope. Viral supernatants containing pseudotyped virus were collected approximately 48 hours post-transfection, purified through 0.22 µm filters and concentrated over 30% sucrose cushions. Concentrated virus was titred for infectivity (TCID_50_) on TZM-bl cells as previously described and stored at −80°C until use [45]. TCID_50_ ranged from 10^4.8^–10^6.2^ for JRFL pseudotyped virions and 10^5.4^–10^6.3^ for VSV-G pseudotyped virions.

### Non-human primate studies

In total 14 female rhesus macaques (*Macaca mulatta; RM*) were used in this study, 5 of which have had one offspring and 8 of which are multiparous. Animals treated with Depo-Provera (DP) were given a single 30 mg dose one month prior to viral inoculation. All animals received a vaginal biopsy in the upper vagina/fornix, and were subsequently atraumatically inoculated vaginally and rectally with virus supernatant. Groups of 6 (2 DP Treated) and 8 RMs (4 DP Treated) were inoculated with VSV-G or JRFL Env expressing virions, respectively. Animals were sacrificed 48 hours after inoculation and their genital tracts were removed, stored in RPMI and shipped on ice overnight for further processing. This timeframe was chosen because it was the shortest time interval in which we could reliably detect reporter expression. Whole reproductive tracts, inguinal, and iliac lymph nodes were washed in PBS, then soaked in 100 mM d-Luciferin (Biosynth) and placed in the IVIS device to examine luciferase expression. Reproductive tract tissue was next divided in 5 regions: labia and lower vagina, upper vagina/fornix, cervix, uterus and ovaries and reimaged. Foci of luminescence detected in the second screen were excised and examined again for luciferase expression. Domains with luciferase detected in the third screen were cut to 2×2 mm^2^ pieces and frozen in optimal cutting temperature (OCT) compound. All analysis of luminescent signals was performed on Living Image Software.

### Nested PCR of mCherry gene

Genomic DNA was isolated from 3 to 5 mg of frozen tissue using the Qaigen DNeasy Blood & Tissue Kit (Qaigen N.V., Valencia, California, USA). In each nested PCR reaction, 250 ng of genomic DNA was utilized to detect a 268-bp DNA fragment of the mCherry reporter gene using an amplification procedure based on previously described methodology [25]. The first round primers were 5′-ATAACATGGCCATCATCAAGGAGT-3′ (forward) and 5′-GTACTGTTCCACGATGGTGTAGTC-3′ (reverse). In the second round, 2 µl of the first round reaction products were amplified with 5′-CCGACTACTTGAAGCTGTCCTT-3′ (forward) and 5′-GTCTTGACCTCAGCGTCGTAGT-3′ (reverse). Amplification was performed using a Bio-Rad iCycler Thermal Cycler system (Bio-Rad Laboratories, Hercules, CA, USA). Each DNA sample was tested in ≥24 replicates. Negative controls were rhesus macaque ovary tissue from animals not exposed to the mCherry containing reporter vector. Second round PCR products were separated on a 1.5% agarose gel and visualized by ethidium bromide staining. Sequences were confirmed by extracting DNA with Qaigen QIAquick Gel Extraction Kit analysis with the second round primers.

### Tissue staining and microscopy

Cryosections of tissue (20 micron thickness) were fixed in 1% formaldehyde in PIPES buffer. To decrease background, tissue was treated with 100 mM L-Lysine. Tissue was stained for luciferase expression with polyclonal rabbit anti-firefly (Abcam), pre-labeled with Xenon AlexaFluor-647 (Invitrogen). Phenotyping target cells was achieved by blocking with donkey serum and subsequently staining with either rabbit anti-human CD3 (clone SP7, Abcam) followed by AlexaFluor488 conjugated donkey anti-rabbit Ig (Jackson Immuno), mouse anti-human CD4 (clone OKT4, hybridoma supernatant) followed by AlexaFluor488 conjugated donkey anti-mouse Ig (Invitrogen), or mouse anti-human CD68 (clone EBM11, Dako) followed by AlexaFluor488 conjugated donkey anti-mouse Ig. Spectral imaging of mCherry expression was performed on a Nikon A1R laser scanning confocal microscope equipped with a 60× objective and Nikon Elements Software. 5 color (DAPI, AlexaFluor488, TRITC, mCherry, AlexaFluor647) image stacks containing 20–40 sections in the Z plane in 0.5 µm steps were acquired and deconvolved using softWoRx software (Applied Precision) on a DeltaVision inverted microscope.

## Supporting Information

Figure S1 **Quantification of luminescent signal in transduced cells transferred to nude mice.** (a) Rhesus macaque PBMCs are infected with JRFL pseudotyped virions, and mCherry expressing cells are selected by cell sorting. mCherry positive cells were defined by fluorescence signal relative to uninfected cells. The gating strategy isolates leukocytes by cell size and ensures cells are singlets and have bright mCherry expression, relative to uninfected controls. (b) Increasing numbers of cells are transferred via subcutaneous injection into nude mice (strain: SKH1), followed by intraperitoneal delivery of 100 mM d-Luciferin. Luminescent flux across the area of injection is measured by *in vivo* imaging system; a representative image of one mouse is shown. (c) Luminescent signal at the site of injection is related to the number of mCherry expressing cells transferred by a power law. Horizontal line indicates limit of detection (LOD), defined by 2.5× background signal. n≥3 for each measurement. Error bars indicate standard error.
(TIF)

Figure S2 **Background luminescence levels from unchallenged macaque.** In vivo imaging analysis of an unchallenged macaque treated with Luciferin was used to define background signal and threshold the luminescence from all LICh inoculated macaques. (Animal code: GA64).
(TIF)

Figure S3 **Infection of PBMC from two Rhesus Macaques with JRFL pseudotyped virions.** 48 hours after infection with vector, infected cells are identified by fluorescence microscopy. Cells express mCherry, and stain positive for luciferase expression and HIV-1 receptor CD4, while remaining dim for TRITC. mCherry, luciferase, or TRITC signal is shown in red. CD4 is shown in green. Nuclear counterstain (DAPI) is shown in blue. (Animal code: (a) EH99, (b) FM27) Scale bars, 10 µm.
(TIF)

Figure S4 **Secondary-only antibody controls are used to set thresholds for specific fluorescent signal.** Tissue sections from the same vaginal vault tissues as shown in Figure 4 were fixed, blocked, and stained with the same methodology, omitting the primary antibody to determine background fluorescence from specific signal. (Animal code: EH99).
(TIF)

Figure S5 **The inguinal lymph node of a JRFL inoculated Rhesus Macaque harbors infected CD4 expressing cells.** Although a high degree of auto-fluorescence limits the scope of tissue examination, some reporter signal distinguishable from background is observed in susceptible cells. mCherry, luciferase, or TRITC signal is shown in red. CD4 is shown in green. Nuclear counterstain (DAPI) is shown in blue. Scale bars, 10.7 µm. (Animal code: FM09).
(TIF)

## References

[1] Joint United Nations Programme on HIV/AIDS. (2010) Global report: UNAIDS report on the global AIDS epidemic. Geneva, Switzerland: Joint United Nations Programme on HIV/AIDS. pp. v.

[2] Haynes BF, McElrath MJ (2013) Progress in HIV-1 vaccine development. Curr Opin HIV AIDS 8: 326–332. 23743722 10.1097/COH.0b013e328361d178 PMC3947525

[3] Shattock RJ, Moore JP (2003) Inhibiting sexual transmission of HIV-1 infection. Nat Rev Microbiol 1: 25–34. 15040177 10.1038/nrmicro729

[4] Haase AT (2011) Early events in sexual transmission of HIV and SIV and opportunities for interventions. Annu Rev Med 62: 127–139. 21054171 10.1146/annurev-med-080709-124959

[5] Keele BF, Estes JD (2011) Barriers to mucosal transmission of immunodeficiency viruses. Blood 118: 839–846. 21555745 10.1182/blood-2010-12-325860 PMC3148165

[6] Stone M, Keele BF, Ma ZM, Bailes E, Dutra J, et al (2010) A limited number of simian immunodeficiency virus (SIV) env variants are transmitted to rhesus macaques vaginally inoculated with SIVmac251. J Virol 84: 7083–7095. 20463069 10.1128/JVI.00481-10 PMC2898254

[7] Miller CJ, Li Q, Abel K, Kim EY, Ma ZM, et al (2005) Propagation and dissemination of infection after vaginal transmission of simian immunodeficiency virus. J Virol 79: 9217–9227. 15994816 10.1128/JVI.79.14.9217-9227.2005 PMC1168785

[8] Li Q, Estes JD, Schlievert PM, Duan L, Brosnahan AJ, et al (2009) Glycerol monolaurate prevents mucosal SIV transmission. Nature 458: 1034–1038. 19262509 10.1038/nature07831 PMC2785041

[9] Grant RM, Lama JR, Anderson PL, McMahan V, Liu AY, et al (2010) Preexposure Chemoprophylaxis for HIV Prevention in Men Who Have Sex with Men. New England Journal of Medicine 363: 2587–2599. 21091279 10.1056/NEJMoa1011205 PMC3079639

[10] Abdool Karim Q, Abdool Karim SS, Frohlich JA, Grobler AC, Baxter C, et al (2010) Effectiveness and safety of tenofovir gel, an antiretroviral microbicide, for the prevention of HIV infection in women. Science 329: 1168–1174. 20643915 10.1126/science.1193748 PMC3001187

[11] J Marrazzo GR, Nair G, et al.. (2013) Pre-exposure prophylaxis for HIV in women: daily oral tenofovir, oral tenofovir/emtricitabine or vaginal tenofovir gel in the VOICE study (MTN 003). Conference on Retroviruses and Opportunistic Infections Goergia World Congress Centre, Atlanta.

[12] Friend DR, Kiser PF (2013) Assessment of topical microbicides to prevent HIV-1 transmission: Concepts, testing, lessons learned. Antiviral Research 99: 391–400. 23845918 10.1016/j.antiviral.2013.06.021

[13] Van Damme L, Corneli A, Ahmed K, Agot K, Lombaard J, et al (2012) Preexposure Prophylaxis for HIV Infection among African Women. N Engl J Med 10.1056/NEJMoa1202614 PMC3687217 22784040

[14] Negre D, Mangeot PE, Duisit G, Blanchard S, Vidalain PO, et al (2000) Characterization of novel safe lentiviral vectors derived from simian immunodeficiency virus (SIVmac251) that efficiently transduce mature human dendritic cells. Gene Ther 7: 1613–1623. 11083469 10.1038/sj.gt.3301292

[15] Shaner NC, Campbell RE, Steinbach PA, Giepmans BN, Palmer AE, et al (2004) Improved monomeric red, orange and yellow fluorescent proteins derived from Discosoma sp. red fluorescent protein. Nat Biotechnol 22: 1567–1572. 15558047 10.1038/nbt1037

[16] Rabinovich BA, Ye Y, Etto T, Chen JQ, Levitsky HI, et al (2008) Visualizing fewer than 10 mouse T cells with an enhanced firefly luciferase in immunocompetent mouse models of cancer. Proc Natl Acad Sci U S A 105: 14342–14346. 18794521 10.1073/pnas.0804105105 PMC2567214

[17] Koyanagi Y, Miles S, Mitsuyasu RT, Merrill JE, Vinters HV, et al (1987) Dual infection of the central nervous system by AIDS viruses with distinct cellular tropisms. Science 236: 819–822. 3646751 10.1126/science.3646751

[18] Cronin J, Zhang XY, Reiser J (2005) Altering the tropism of lentiviral vectors through pseudotyping. Curr Gene Ther 5: 387–398. 16101513 10.2174/1566523054546224 PMC1368960

[19] Zufferey R, Donello JE, Trono D, Hope TJ (1999) Woodchuck hepatitis virus posttranscriptional regulatory element enhances expression of transgenes delivered by retroviral vectors. J Virol 73: 2886–2892. 10074136 10.1128/jvi.73.4.2886-2892.1999 PMC104046

[20] Donello JE, Loeb JE, Hope TJ (1998) Woodchuck hepatitis virus contains a tripartite posttranscriptional regulatory element. J Virol 72: 5085–5092. 9573279 10.1128/jvi.72.6.5085-5092.1998 PMC110072

[21] Durand S, Cimarelli A (2011) The inside out of lentiviral vectors. Viruses 3: 132–159. 22049307 10.3390/v3020132 PMC3206600

[22] Marx PA, Spira AI, Gettie A, Dailey PJ, Veazey RS, et al (1996) Progesterone implants enhance SIV vaginal transmission and early virus load. Nat Med 2: 1084–1089. 8837605 10.1038/nm1096-1084

[23] Lee B, Sharron M, Montaner LJ, Weissman D, Doms RW (1999) Quantification of CD4, CCR5, and CXCR4 levels on lymphocyte subsets, dendritic cells, and differentially conditioned monocyte-derived macrophages. Proc Natl Acad Sci U S A 96: 5215–5220. 10220446 10.1073/pnas.96.9.5215 PMC21844

[24] Poonia B, Walter L, Dufour J, Harrison R, Marx PA, et al (2006) Cyclic changes in the vaginal epithelium of normal rhesus macaques. J Endocrinol 190: 829–835. 17003283 10.1677/joe.1.06873

[25] Milush JM, Kosub D, Marthas M, Schmidt K, Scott F, et al (2004) Rapid dissemination of SIV following oral inoculation. AIDS 18: 2371–2380. 15622313

[26] Zhang Z, Schuler T, Zupancic M, Wietgrefe S, Staskus KA, et al (1999) Sexual transmission and propagation of SIV and HIV in resting and activated CD4+ T cells. Science 286: 1353–1357. 10558989 10.1126/science.286.5443.1353

[27] Miller CJ, Shattock RJ (2003) Target cells in vaginal HIV transmission. Microbes Infect 5: 59–67. 12593974 10.1016/s1286-4579(02)00056-4

[28] Spira AI, Marx PA, Patterson BK, Mahoney J, Koup RA, et al (1996) Cellular targets of infection and route of viral dissemination after an intravaginal inoculation of simian immunodeficiency virus into rhesus macaques. J Exp Med 183: 215–225. 8551225 10.1084/jem.183.1.215 PMC2192425

[29] Hladik F, Sakchalathorn P, Ballweber L, Lentz G, Fialkow M, et al (2007) Initial events in establishing vaginal entry and infection by human immunodeficiency virus type-1. Immunity 26: 257–270. 17306567 10.1016/j.immuni.2007.01.007 PMC1885958

[30] Pandrea I, Parrish NF, Raehtz K, Gaufin T, Barbian HJ, et al (2012) Mucosal simian immunodeficiency virus transmission in African green monkeys: susceptibility to infection is proportional to target cell availability at mucosal sites. J Virol 86: 4158–4168. 22318138 10.1128/JVI.07141-11 PMC3318646

[31] Wira CR, Fahey JV (2008) A new strategy to understand how HIV infects women: identification of a window of vulnerability during the menstrual cycle. AIDS 22: 1909–1917. 18784454 10.1097/QAD.0b013e3283060ea4 PMC2647143

[32] Cramer DW, Welch WR, Berkowitz RS, Godleski JJ (2007) Presence of talc in pelvic lymph nodes of a woman with ovarian cancer and long-term genital exposure to cosmetic talc. Obstetrics and gynecology 110: 498–501. 17666642 10.1097/01.AOG.0000262902.80861.a0

[33] Rosenblatt KA, Weiss NS, Cushing-Haugen KL, Wicklund KG, Rossing MA (2011) Genital powder exposure and the risk of epithelial ovarian cancer. Cancer Causes Control 22: 737–742. 21516319 10.1007/s10552-011-9746-3 PMC3384556

[34] Cook LS, Kamb ML, Weiss NS (1997) Perineal powder exposure and the risk of ovarian cancer. Am J Epidemiol 145: 459–465. 9048520 10.1093/oxfordjournals.aje.a009128

[35] Blumenkrantz MJ, Gallagher N, Bashore RA, Tenckhoff H (1981) Retrograde menstruation in women undergoing chronic peritoneal dialysis. Obstetrics and gynecology 57: 667–670. 7219918

[36] Halme J, Hammond MG, Hulka JF, Raj SG, Talbert LM (1984) Retrograde menstruation in healthy women and in patients with endometriosis. Obstetrics and gynecology 64: 151–154. 6234483

[37] Masurier C, Salomon B, Guettari N, Pioche C, Lachapelle F, et al (1998) Dendritic cells route human immunodeficiency virus to lymph nodes after vaginal or intravenous administration to mice. J Virol 72: 7822–7829. 9733818 10.1128/jvi.72.10.7822-7829.1998 PMC110098

[38] Ghanem KG, Shah N, Klein RS, Mayer KH, Sobel JD, et al (2005) Influence of sex hormones, HIV status, and concomitant sexually transmitted infection on cervicovaginal inflammation. J Infect Dis 191: 358–366. 15633094 10.1086/427190

[39] Rotchford K, Strum AW, Wilkinson D (2000) Effect of coinfection with STDs and of STD treatment on HIV shedding in genital-tract secretions: systematic review and data synthesis. Sex Transm Dis 27: 243–248. 10821594 10.1097/00007435-200005000-00001

[40] Vishwanathan SA, Guenthner PC, Lin CY, Dobard C, Sharma S, et al (2011) High susceptibility to repeated, low-dose, vaginal SHIV exposure late in the luteal phase of the menstrual cycle of pigtail macaques. J Acquir Immune Defic Syndr 57: 261–264. 21546848 10.1097/QAI.0b013e318220ebd3

[41] Kersh EN, Henning T, Vishwanathan SA, Morris M, Butler K, et al (2014) SHIV susceptibility changes during the menstrual cycle of pigtail macaques. J Med Primatol [epub ahead of print].. 10.1111/jmp.12124 PMC4175065 24779484

[42] Akiyama M, Okabe H, Takakura K, Fujiyama Y, Noda Y (1999) Expression of macrophage inflammatory protein-1alpha (MIP-1alpha) in human endometrium throughout the menstrual cycle. Br J Obstet Gynaecol 106: 725–730. 10428531 10.1111/j.1471-0528.1999.tb08374.x

[43] Kallikourdis M, Andersen KG, Welch KA, Betz AG (2007) Alloantigen-enhanced accumulation of CCR5+ ‘effector’ regulatory T cells in the gravid uterus. Proc Natl Acad Sci U S A 104: 594–599. 17197426 10.1073/pnas.0604268104 PMC1766430

[44] Padian NS, van der Straten A, Ramjee G, Chipato T, de Bruyn G, et al (2007) Diaphragm and lubricant gel for prevention of HIV acquisition in southern African women: a randomised controlled trial. Lancet 370: 251–261. 17631387 10.1016/S0140-6736(07)60950-7 PMC2442038

[45] Wei X, Decker JM, Liu H, Zhang Z, Arani RB, et al (2002) Emergence of resistant human immunodeficiency virus type 1 in patients receiving fusion inhibitor (T-20) monotherapy. Antimicrob Agents Chemother 46: 1896–1905. 12019106 10.1128/AAC.46.6.1896-1905.2002 PMC127242

