# Supplementary figures and images for: Vaginal Challenge with an SIV-Based Dual Reporter System Reveals That Infection Can Occur throughout the Upper and Lower Female Reproductive Tract
Source: PLoS Pathog. 2014 Oct 9;10(10):e1004440. doi: 10.1371/journal.ppat.1004440 (PMC4192600; doi:10.1371/journal.ppat.1004440)

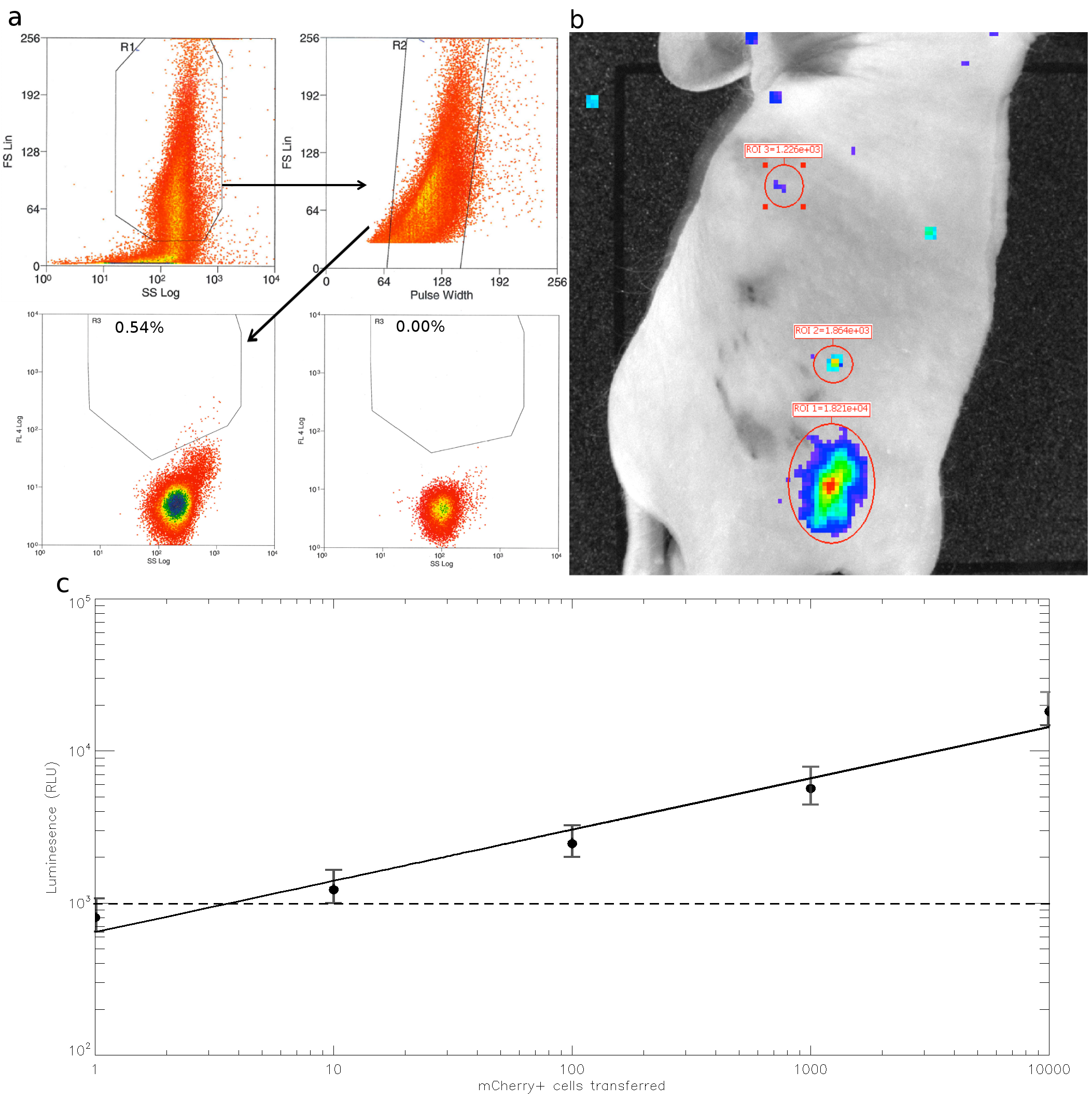

Supplement: Figure S1 — Quantification of luminescent signal in transduced cells transferred to nude mice. (a) Rhesus macaque PBMCs are infected with JRFL pseudotyped virions, and mCherry expressing cells are selected by cell sorting. mCherry positive cells were defined by fluorescence signal relative to uninfected cells. The gating strategy isolates leukocytes by cell size and ensures cells are singlets and have bright mCherry expression, relative to uninfected controls. (b) Increasing numbers of cells are transferred via subcutaneous injection into nude mice (strain: SKH1), followed by intraperitoneal delivery of 100 mM d-Luciferin. Luminescent flux across the area of injection is measured by in vivo imaging system; a representative image of one mouse is shown. (c) Luminescent signal at the site of injection is related to the number of mCherry expressing cells transferred by a power law. Horizontal line indicates limit of detection (LOD), defined by 2.5× background signal. n≥3 for each measurement. Error bars indicate standard error. (TIF) [file ppat.1004440.s001.tif]

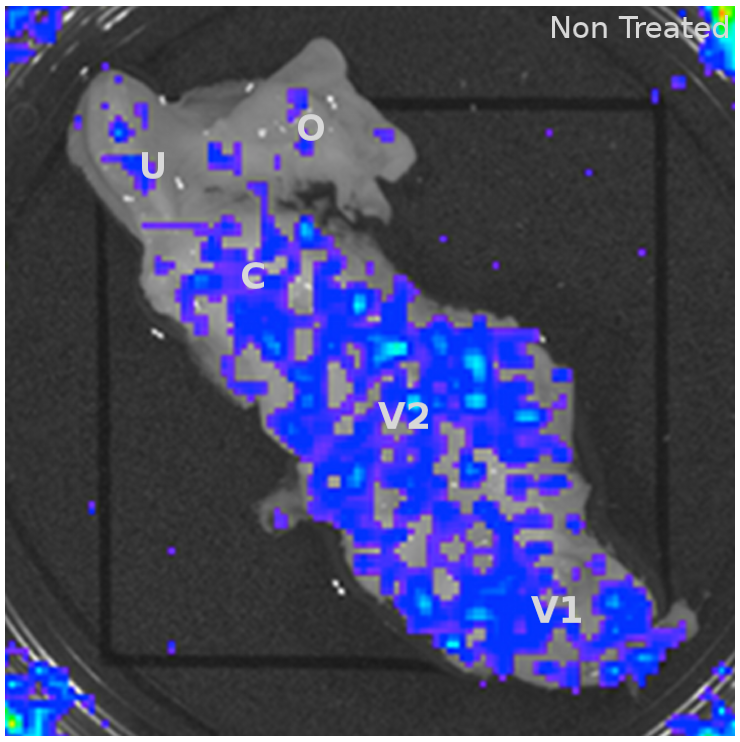

Supplement: Figure S2 — Background luminescence levels from unchallenged macaque. In vivo imaging analysis of an unchallenged macaque treated with Luciferin was used to define background signal and threshold the luminescence from all LICh inoculated macaques. (Animal code: GA64). (TIF) [file ppat.1004440.s002.tif]

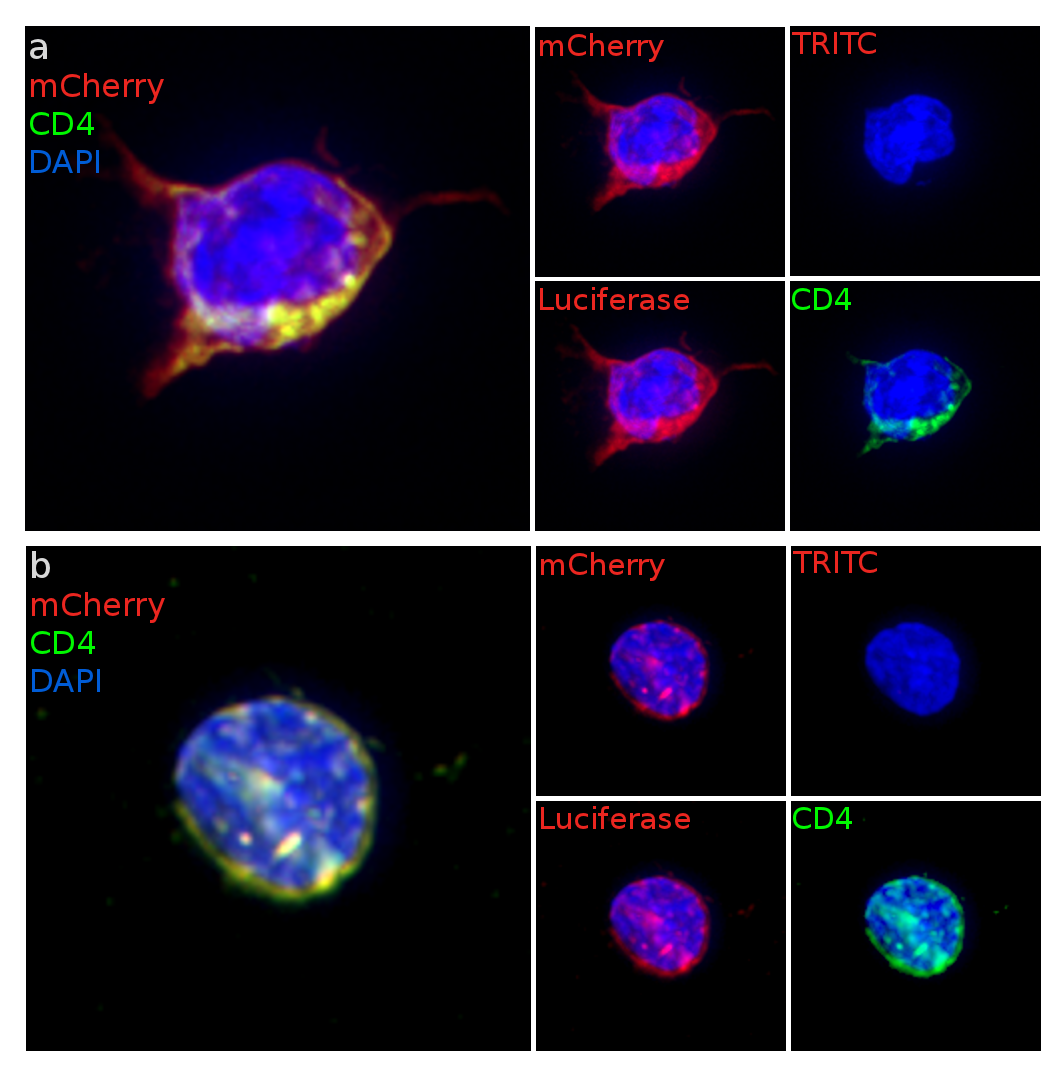

Supplement: Figure S3 — Infection of PBMC from two Rhesus Macaques with JRFL pseudotyped virions. 48 hours after infection with vector, infected cells are identified by fluorescence microscopy. Cells express mCherry, and stain positive for luciferase expression and HIV-1 receptor CD4, while remaining dim for TRITC. mCherry, luciferase, or TRITC signal is shown in red. CD4 is shown in green. Nuclear counterstain (DAPI) is shown in blue. (Animal code: (a) EH99, (b) FM27) Scale bars, 10 µm. (TIF) [file ppat.1004440.s003.tif]

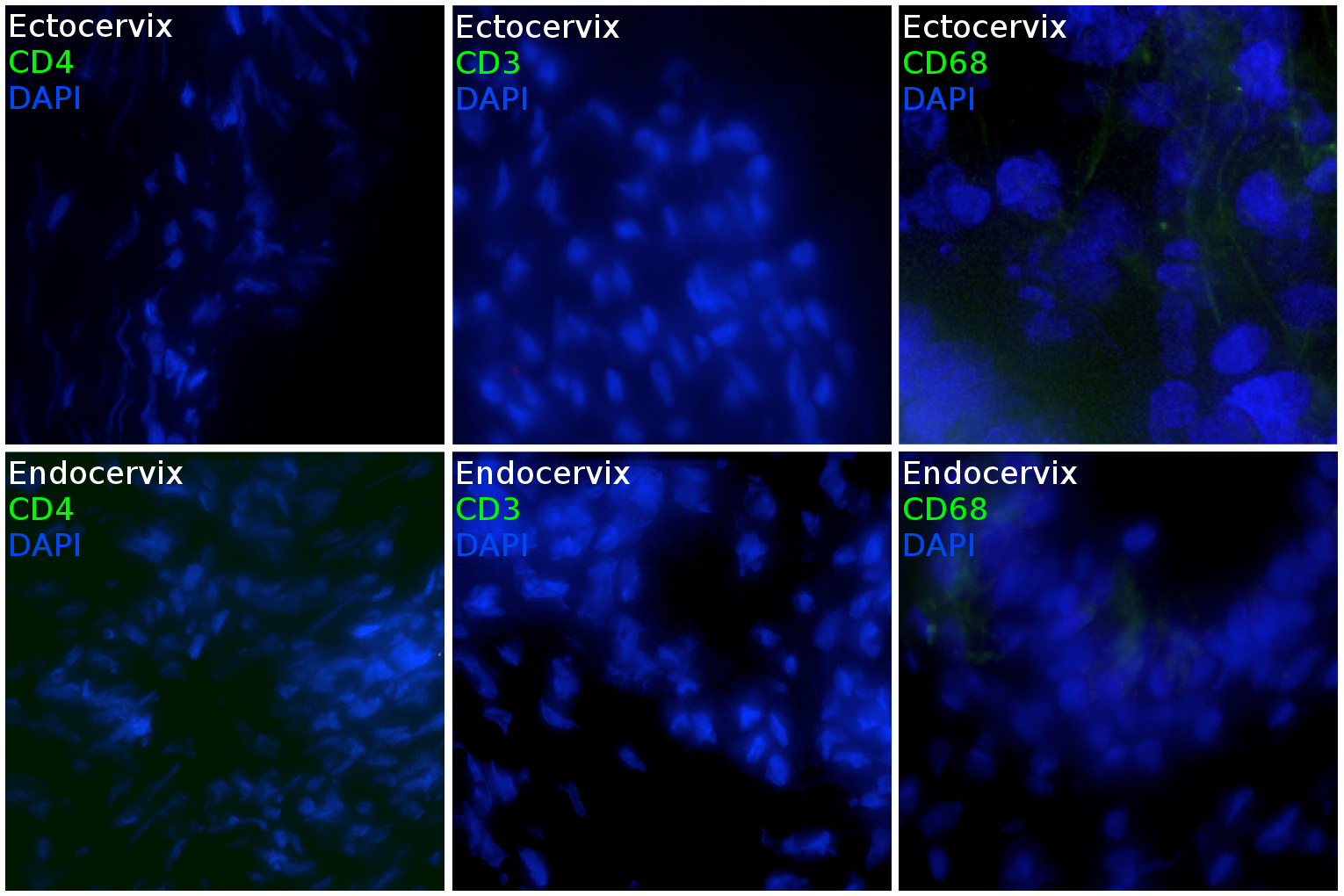

Supplement: Figure S4 — Secondary-only antibody controls are used to set thresholds for specific fluorescent signal. Tissue sections from the same vaginal vault tissues as shown in Figure 4 were fixed, blocked, and stained with the same methodology, omitting the primary antibody to determine background fluorescence from specific signal. (Animal code: EH99). (TIF) [file ppat.1004440.s004.tif]

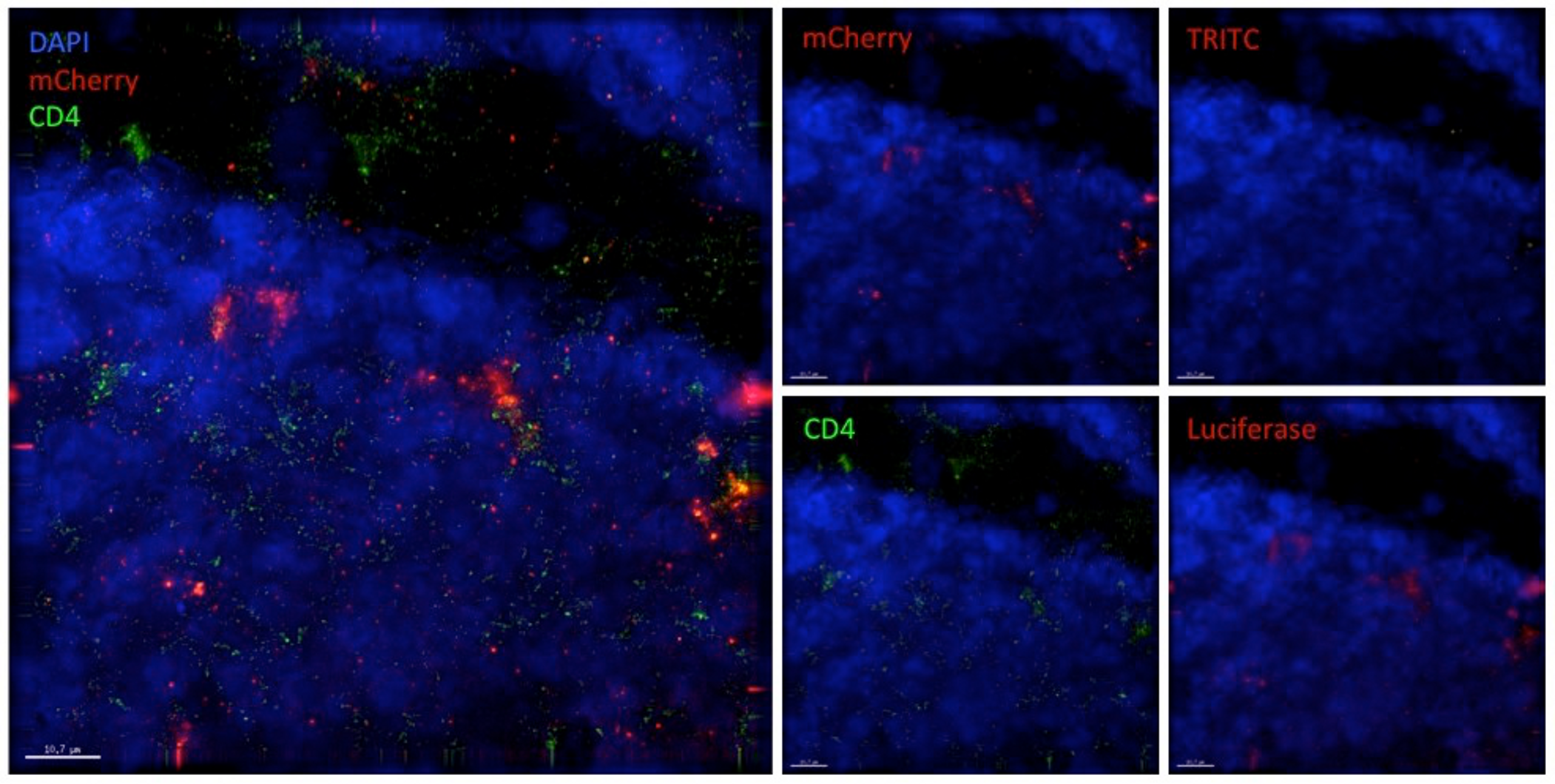

Supplement: Figure S5 — The inguinal lymph node of a JRFL inoculated Rhesus Macaque harbors infected CD4 expressing cells. Although a high degree of auto-fluorescence limits the scope of tissue examination, some reporter signal distinguishable from background is observed in susceptible cells. mCherry, luciferase, or TRITC signal is shown in red. CD4 is shown in green. Nuclear counterstain (DAPI) is shown in blue. Scale bars, 10.7 µm. (Animal code: FM09). (TIF) [file ppat.1004440.s005.tif]
